# Optimizing rangeland use: Forage selection and grazing patterns of Nguni and Bonsmara cattle across traditional and commercial systems

**DOI:** 10.1016/j.vas.2025.100436

**Published:** 2025-03-06

**Authors:** Mhlangabezi Slayi, Ishmael Festus Jaja

**Affiliations:** a Centre for Global Change (CGC), University of Fort Hare, Dikeni, South Africa; b Department of Livestock and Pasture Science, University of Fort Hare, Dikeni, South Africa

**Keywords:** Breed adaptation, Climatic variability, Pasture quality, Grazing behavior, Forage utilization

## Abstract

This study examined the grazing dynamics of Nguni and Bonsmara cattle across traditional communal and commercial ranching systems in semi-arid South Africa. Eighty cattle (*n* = 20 per breed and management system) were monitored over 12 months to assess grazing behavior, forage selection, and environmental interactions. Data collection involved direct behavioral observations, GPS tracking of movement patterns, and forage quality assessments across seasons. Significant breed-specific differences were observed, with Nguni cattle exhibiting longer grazing durations (*p* = 0.02), higher step rates (*p* = 0.03), and broader dietary flexibility (*p* = 0.04) in communal systems, enabling efficient utilization of lower-quality forage. Conversely, Bonsmara cattle, optimized for commercial production, displayed a preference for high-quality forage and engaged in shorter, more concentrated grazing bouts (*p* = 0.01), leading to superior weight gain (*p* = 0.03) in nutrient-rich pastures. Seasonal fluctuations significantly influenced grazing behavior, with reduced forage availability during the dry season disproportionately affecting Bonsmara cattle in communal areas (*p* = 0.02). These findings underscore the importance of breed selection and adaptive grazing management for optimizing livestock productivity and sustainability across diverse agroecological systems.

## 1 Introduction

Livestock production is a cornerstone of rural livelihoods and food security in semi-arid regions of South Africa, where extensive rangeland grazing remains the dominant practice (Mapfumo et al., 2017; Fayemi & Muchenje, 2012; Myburgh et al., 2012; Napolitano et al., 2011). Cattle farming not only provides economic sustenance but also holds cultural significance, serving as a key asset in wealth accumulation and risk mitigation for rural households ( Moya et al., 2014; Mezzalira et al., 2012; Mapiye et al., 2019). However, the sustainability of cattle production in these regions is increasingly threatened by climate variability, land degradation, and competition for limited resources (Mapiye et al., 2020; Lamy et al., 2012; Kelly et al., 2010). These challenges necessitate adaptive management strategies that enhance both livestock productivity and rangeland health (Katiyatiya et al., 2017; Matlebyane et al., 2009; McDonnell et al., 2016). Nguni and Bonsmara cattle are among the most widely utilized breeds for beef production in South Africa (Slayi et al., 2021; Scholtz et al., 2013), yet they differ significantly in their genetic adaptations and suitability for various management systems (Slayi et al., 2022; Muchenje et al., 2008). Nguni cattle, indigenous to southern Africa, exhibit resilience to heat stress, parasites, and nutritional fluctuations, making them well-suited for communal grazing systems that depend on resource-sharing and minimal external inputs (Slayi et al., 2023; Fayemi & Muchenje, 2012; Calus et al., 2023; Cavallini et al., 2023). In contrast, Bonsmara cattle, selectively bred for superior growth rates and meat quality, perform optimally in commercial ranching systems where controlled grazing, supplementation, and intensive herd management enhance productivity (Lawrence et al., 2012; Meyer et al., 2008;Trindade et al., 2012). While previous research has extensively documented the production characteristics of these breeds, there remains a significant knowledge gap in understanding their comparative grazing behaviors and forage utilization patterns under distinct management systems.

Grazing dynamics, encompassing bite rate, step rate, forage selection, and spatial distribution are critical in determining livestock productivity and the sustainability of rangeland ecosystems (Linde, 2018; Myburgh et al., 2012; Napolitano et al., 2011). Breed-specific foraging strategies, combined with environmental stressors such as drought and overgrazing, influence grazing efficiency, diet composition, and overall adaptation to specific management conditions (Nyamushamba et al., 2017; Williams et al., 2016; Cantalapiedra-Hijar et al., 2018). Despite an increasing focus on sustainable rangeland management, few studies have systematically compared how Nguni and Bonsmara cattle interact with their grazing environments, particularly in the context of communal versus commercial systems. This study addresses this gap by evaluating the grazing dynamics of Nguni and Bonsmara cattle under different management systems, with a focus on forage utilization, behavioral patterns, and the influence of environmental factors on grazing efficiency. By identifying breed-specific adaptations and management implications, the study provides actionable insights to optimize cattle production while promoting the long-term sustainability of rangeland resources in semi-arid regions of South Africa. Future research should expand on these findings by exploring additional environmental variables and assessing the long-term impacts of grazing strategies on rangeland health.

## 2 Materials and methods

### 2.1 Ethical clearance statement

The study was conducted in compliance with the University of Fort Hare's Research Ethics Policy regarding the housing and treatment of animals. The research protocol underwent thorough review and received approval under the ethical clearance certificate number JAJ051SMPO01 from the institutional Animal Research Ethics Committee.

### 2.2 Study site

The study was conducted in semi-arid regions of South Africa, characterized by mixed grass species (e.g., Panicum maximum, Themeda triandra) and varied seasonal precipitation. The research took place in Alice, within the Raymond Mhlaba Local Municipality, representing the traditional communal grazing system, and at the Bathurst Research Station, within the Sarah Baartman District Municipality, representing the commercial grazing system. These locations are both in the Eastern Cape region of South Africa and were deliberately chosen for their distinct ecological and management characteristics. Alice is positioned at 32° 8′ Longitude and 26° 85′ S Latitude, with an elevation of 500 m above sea level. The region receives an annual rainfall of 480 mm and has an average temperature of 18.7 °C (Eastern Cape Socio-Economic Consultative Council, 2012). Alice falls within the Bisho Thornveld, with vegetation primarily consisting of diverse grass species such as *Digitaria eriantha, Aristida congesta, Cynodon dactylon, Eragrostis* spp., *Sporobolus fimbriatus, Themeda triandra*, and *Sporobolus africanus*. Dominant tree species in the area include *Maytenus polyacantha, Scutia myrtina, and Acacia karroo* (Mucina & Rutherford, 2011). The traditional grazing system in Alice is characterized by communal land tenure, where livestock graze freely across shared rangelands with minimal intervention in terms of rotational grazing or supplementary feeding (Mezzalira et al., 2012; Asizua, 2010; Moya et al., 2014). Cattle are managed extensively by multiple households, leading to continuous grazing pressure and competition for forage resources (Trindade et al., 2012; Williams et al., 2016). Water availability is dependent on natural sources such as rivers and seasonal water pans (Fitzsimons et al., 2014; Fitzsimons et al., 2013, Hardy and Walker, 1991). Due to high stocking densities and limited grazing management, rangeland degradation is a common concern, influencing forage availability and quality (Lamy et al., 2012; Lawrence et al., 2012; Linde, 2018).

Bathurst Research Station is situated within the Kowie Thicket biome, at 33° 30′ S latitude and 26° 49′ E longitude, with an elevation of 708 m above sea level. This location experiences an annual rainfall of 624 mm, with summer temperatures ranging from 13 to 29 °C and winter temperatures from 1 to 12 °C (Acocks, 1988). The vegetation is characterized by tall thickets dominated by succulent aloes and euphorbias, with a thick understory comprising woody lianas (*Capparis, Rhoicissus, Aloe, Secamone*), shrubby succulents (*Crassulaceae, Asphodelaceae*), and thorny shrubs. The moist south-facing slopes support dense thickets of evergreen trees (*Euclea, Pappea, Cussonia, Ptaeroxylon, Hippobromus, Schotia*) and shrubs (*Putterlickia, Gymnosporia, Carissa, Azima*), where the herbaceous layer is sparse due to low light penetration (Nciizha & Wakindiki, 2012). In contrast to Alice, the commercial grazing system at Bathurst Research Station operates under structured pasture management with controlled stocking rates, rotational grazing, and supplementary feeding when necessary. The land is privately managed, allowing for greater oversight of grazing intensity and forage conservation (Casey, 2021, Musemwa et al., 2012). Artificial water points and controlled breeding programs further distinguish this system, ensuring optimal cattle productivity while minimizing environmental degradation (Jochims et al., 2020; Jorge-Smeding et al., 2021). These two locations provide a unique juxtaposition of traditional and commercial grazing practices within a relatively small geographic area. The ecological and climatic variability between Alice and Bathurst allows for a comprehensive assessment of how different management approaches impact cattle adaptation, nutritional intake, behavior, rumen fermentation, and overall performance. The inclusion of both systems ensures a well-rounded understanding of cattle production dynamics under varying grazing conditions, offering valuable insights into sustainable rangeland management and livestock productivity in semi-arid regions (Owens et al., 2009, Singaravadivelan et al., 2023).

### 2.3 Study design and selection of experimental animals

A total of 80 cattle (40 Nguni and 40 Bonsmara) were selected and stratified by breed, sex, and body weight to ensure comparability. Each group was equally distributed across traditional and commercial grazing systems. Prior to inclusion in the study, all animals underwent a comprehensive health screening conducted by an experienced veterinarian to ensure homogeneity within the cohort. Health assessments included body condition scoring, disease screening, and overall fitness evaluation. To minimize stress and facilitate adaptation to study conditions, all cattle underwent a one-month acclimatization period before data collection. The study design followed a proportional observational approach over 12 months, capturing seasonal variations in grazing patterns and environmental influences. The animals, all of similar age (18 months) and physiological status (non-lactating, mature females), were systematically allocated into four groups of ten per breed. Each group was assigned to either traditional or commercial grazing systems, with trained personnel conducting daily monitoring to assess health, behavior, and any signs of distress. This rigorous selection and monitoring process ensured that the study accurately captured the effects of breed (Nguni vs. Bonsmara) and grazing system (traditional vs. commercial) on dry matter intake, weight gain, and behavioral patterns. The stratification process accounted for key variables affecting grazing efficiency and performance, providing a robust framework for evaluating the impact of different management practices on the two cattle breeds.

### 2.4 Data collection

#### 2.4.1 Behavioral observations and daily forage consumption estimation

The study was conducted over a full year to account for seasonal variations in forage availability, climatic conditions, and their influence on ingestive behavior. Observations were carried out continuously across all four seasons (summer, autumn, winter, and spring), ensuring that data captured the effects of fluctuating temperature, precipitation, and forage quality on cattle behavior. Cattle were observed for 15 h daily (5:00AM to 8:00PM), covering morning, midday, and late afternoon periods to document potential diurnal variations in behavior. Observers recorded the time spent in each activity every 15 min using a structured ethogram. To enhance data accuracy and consistency, ten trained observers, each with over three years of experience in behavioral data collection, underwent inter-observer reliability training prior to the study. A standardized data collection protocol was implemented throughout the year to minimize bias and ensure consistency across all seasons. Behavioral observations followed a scan sampling procedure (Cavallini et al., 2023; Slayi et al., 2021), focusing on key activities such as grazing (mouth in contact with grass), drinking (head down with mouth in contact with water), resting (body in contact with the ground in lateral or sternal recumbency), and walking (moving with head raised from one location to another). To facilitate individual identification, cattle were fitted with uniquely numbered and colored ear tags (green for Bonsmara, white for Nguni) and marked with washable dye codes. Since forage availability and quality fluctuate across seasons, a comprehensive forage sampling protocol was implemented to assess nutritional composition. Monthly forage samples were collected from both grazing systems (traditional and commercial) using a quadrant sampling technique. Fresh and dried biomass samples were analyzed for crude protein (CP), neutral detergent fiber (NDF), acid detergent fiber (ADF), and in vitro digestibility. These chemical analyses provided a detailed understanding of the nutritional resources available to cattle and their potential influence on intake and feeding behavior. Daily forage consumption was estimated based on observed grazing time, bite rate, and bite size using the following formula:

DailyForageIntake(kg)=GrazingTime(min)×BiteRate(bites/min)×BiteSize(kg/bite)

Forage digestibility was determined through in vitro dry matter digestibility (IVDMD) assays. The daily forage intake was then adjusted for digestibility to estimate the effective dry matter intake (DMI):

EffectiveDMI(kg/day)=DailyForageIntake(kg)×DigestibilityCoefficient

#### 2.4.2 Environmental data

##### 2.4.2.1 Pasture quality

Forage biomass and stocking rates were assessed seasonally. Chemical composition analyses were conducted using Near-Infrared Reflectance Spectroscopy (NIRS) to evaluate variations in nutritional quality over time. Biomass sampling followed a quadrat method, where forage within a defined 1m² quadrat was clipped at ground level and weighed. Dried samples were analyzed for crude protein, fiber, and other essential nutrients.

##### 2.4.2.2 Water availability

The distance from grazing areas to water sources was measured using GPS tools. This measurement was taken to evaluate how far cattle had to travel to access water. The frequency of cattle visits to water sources was recorded. Observers noted the number of times cattle visited the water sources during the observation periods. This data was used to infer the availability and adequacy of water supply in each grazing system.

##### 2.4.2.3 Weather conditions

Weather stations installed in each grazing system recorded temperature, humidity, and rainfall at hourly intervals. Data analyses included correlations between weather variables and grazing behavior. Extreme weather events, such as heatwaves or heavy rainfall, were documented due to their potential impact on cattle behavior.

#### 2.4.3 Weight gain data collection

All cattle were weighed at baseline and then monthly to monitor weight gain. Initial weights were recorded following a 3-hour fasting period to standardize measurements. Weighing was conducted using a calibrated digital scale (CAUDURO 40 100–1500 kg, Cachoeira do Sul, Brazil) with a precision of ±0.5 kg. The monthly weighing schedule ensured the detection of trends in weight gain linked to grazing system and environmental influences.

$$\text{Average}\;\text{Daily}\;\text{Gain}\;\left(\text{ADG}\right)\;\left(\text{kg}/\text{day}\right)=\frac{Final\;Weight\;\left(kg\right)-Initial\;Weight\;\left(kg\right)}{\text{Number}\;\text{of}\;\text{days}}$$

With DMI and weight gain data available, Forage Conversion Efficiency (FCE) was calculated for each individual:

$$\text{FCE}=\frac{ADG\;\left(kg/day\right)}{DMI\;(kg/day}$$

### 2.5 Statistical analysis

All analyses were conducted using R software (version 3.4.2), with the lme4 package for mixed-effects modeling and the emmeans package for post-hoc tests. Behavioral data were analyzed using a repeated measures ANOVA to assess the effects of breed, grazing system, and seasonality on activity patterns. Pairwise comparisons were conducted using Tukey's post hoc test. Weight gain and FCE were analyzed using linear mixed models, with breed and grazing system as fixed factors and individual animals as random effects. Environmental parameters were included as covariates where applicable. Statistical significance was set at *P* < 0.05. Heatmaps were generated to visualize correlations between forage quality, grazing behavior, and environmental factors. Pooled standard error of the mean (SEM) was included in all tables.

## 3 Results

### 3.1 Grazing patterns of Nguni and Bonsmara cattle in different ranching systems

Table 1 presents the daily grazing patterns of Nguni and Bonsmara cattle under commercial and traditional grazing systems. Nguni cattle spend significantly more time grazing compared to Bonsmara cattle (*p* = 0.03). Although grazing time is higher in commercial systems for both breeds, the difference is not statistically significant (*p* = 0.08). Bonsmara cattle rest significantly more than Nguni cattle (*p* = 0.02). Resting time is slightly higher in traditional systems for both breeds, but this difference is not statistically significant (*p* = 0.06). Nguni cattle have significantly different walking times compared to Bonsmara cattle (*p* = 0.05). Walking time is significantly affected by the grazing system, being higher in traditional systems (*p* = 0.04). Differences in drinking time between breeds and grazing systems are not statistically significant.

**Table 1:** Daily grazing patterns of Nguni and Bonsmara cattle in different ranching systems.

Behavior Variable	Breed	Commercial (%)	Traditional (%)	*p*-value (Breed)	*p*-value (Grazing System)	*p*-value (Breed × Grazing System)
Grazing Time	Nguni	54.1 ± 2.7^a^	51.5 ± 2.3^a^	0.03	0.08	0.11
	Bonsmara	49.3 ± 2.2^b^	47.2 ± 1.8^b^			
Resting Time	Nguni	39.1 ± 1.8^a^	42.0 ± 1.6^b^	0.02	0.06	0.09
	Bonsmara	47.2 ± 1.6^b^	46.0 ± 2.1^b^			
Walking Time	Nguni	6.8 ± 0.6^a^	8.5 ± 1.1^b^	0.05	0.04	0.07
	Bonsmara	7.7 ± 0.5^ab^	7.1 ± 0.6^ab^			
Drinking Time	Nguni	3.7 ± 0.4^a^	3.1 ± 0.3^a^	0.07	0.09	0.12
	Bonsmara	2.8 ± 0.3^b^	4.2 ± 0.6^c^			

*Different superscripts (a, b, c) within rows indicate significant differences (*P* < 0.05).

### 3.2 Forage preference and performance characteristics of Nguni and Bonsmara cattle

**Fig. 1:**
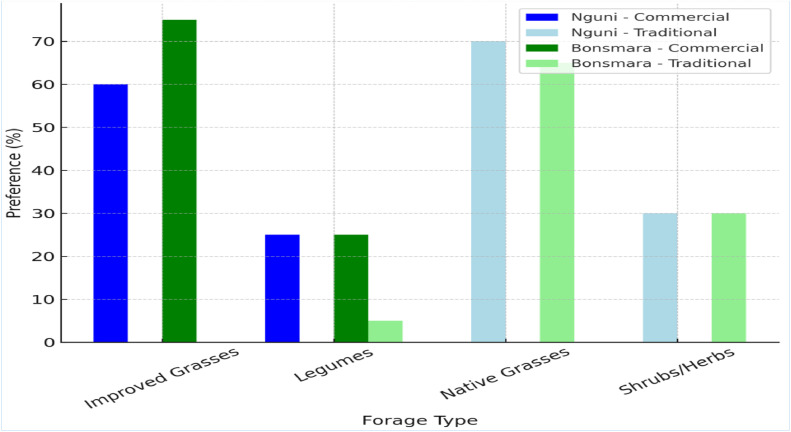
Forage preferences of Nguni and Bonsmara cattle.

Fig. 1 presents detailed forage preferences of Nguni and Bonsmara cattle across different grazing systems. In commercial systems, Nguni cattle predominantly preferred improved grasses such as *Cenchrus ciliaris* (buffelgrass) and *Panicum maximum* (Guinea grass) (60 %), alongside legumes like *Medicago sativa* (alfalfa) and *Stylosanthes* spp. (25 %). In traditional systems, they favored native grasses such as *Themeda triandra* (red grass) and *Eragrostis curvula* (weeping lovegrass) (70 %), along with shrubs and herbs (30 %). Bonsmara cattle in commercial systems showed a stronger preference for improved grasses (75 %), particularly *Cenchrus ciliaris and Digitaria eriantha* (smuts finger grass), and legumes (25 %). In traditional systems, their diet consisted primarily of native grasses (65 %), shrubs and herbs (30 %), with a minor intake of legumes (5 %). The differences in forage type preference were significant between breeds (*p* = 0.04) and grazing systems (*p* = 0.03), though the breed × grazing system interaction was not significant (*p* = 0.06).

Table 2 presents dry matter intake (DMI), forage conversion efficiency (FCE), and weight gain for Nguni and Bonsmara cattle under commercial and traditional grazing systems. For dry matter intake (DMI), Nguni cattle in commercial systems consumed 9.5 ± 0.5 kg/day, while in traditional systems, they consumed 8.0 ± 0.4 kg/day. Bonsmara cattle had a higher intake, with 10.2 ± 0.6 kg/day in commercial systems and 9.0 ± 0.5 kg/day in traditional systems. The differences in DMI were statistically significant between breeds (*p* = 0.03) and grazing systems (*p* = 0.05), although the interaction effect was not significant (*p* = 0.08). Regarding forage conversion efficiency (FCE), Nguni cattle achieved 0.15 ± 0.01 kg gain/kg DMI in commercial systems and 0.12 ± 0.01 kg gain/kg DMI in traditional systems. Bonsmara cattle exhibited higher efficiency, with 0.17 ± 0.01 kg gain/kg DMI in commercial systems and 0.13 ± 0.01 kg gain/kg DMI in traditional systems. These differences were significant for breed (*p* = 0.02) and grazing system (*p* = 0.04), but not for the interaction (*p* = 0.09). For weight gain, Nguni cattle gained 0.85 ± 0.05 kg/day in commercial systems and 0.70 ± 0.04 kg/day in traditional systems. Bonsmara cattle showed higher gains, with 0.95 ± 0.06 kg/day in commercial systems and 0.80 ± 0.05 kg/day in traditional systems. The differences in weight gain were significant for breed (*p* = 0.04) and grazing system (*p* = 0.03), but the interaction between breed and grazing system was not significant (*p* = 0.06).

**Table 2:** Dry matter intake (DMI), forage conversion efficiency (FCE), and weight gain for Nguni and Bonsmara cattle under commercial and traditional grazing systems.

Parameter	Breed	Commercial	Traditional	*p*-value (Breed)	*p*-value (Grazing System)	*p*-value (Breed × Grazing System)
Dry Matter Intake (DMI) (kg/day)	Nguni	9.5 ± 0.5^a^	8.0 ± 0.4^b^	0.03	0.05	0.08
	Bonsmara	10.2 ± 0.6^a^	9.0 ± 0.5^b^			
Forage Conversion Efficiency (FCE) (kg gain/kg DMI)	Nguni	0.15 ± 0.01^a^	0.12 ± 0.01^b^	0.02	0.04	0.09
	Bonsmara	0.17 ± 0.01^a^	0.13 ± 0.01^b^			
Weight Gain (kg/day)	Nguni	0.85 ± 0.05^a^	0.70 ± 0.04^b^	0.04	0.03	0.06
	Bonsmara	0.95 ± 0.06^a^	0.80 ± 0.05^b^			

*Different superscripts (a, b, c) within rows indicate significant differences (*P* < 0.05).

### 3.3 Influence of environmental factors on grazing behavior of Nguni and Bonsmara cattle

Table 3 shows the quality of pasture significantly influenced the grazing behavior of both Nguni and Bonsmara cattle. When provided with high-quality pasture, Nguni cattle spent a significantly greater proportion of their time grazing (61.5 %) compared to when they were on low-quality pasture (50.3 %) (*p* = 0.007). Similarly, Bonsmara cattle showed a decrease in grazing time from 57.8 % on high-quality pasture to 47.5 % on low-quality pasture. The interaction between breed and pasture quality was significant (*p* = 0.032), indicating that the effect of pasture quality on grazing behavior varied between the two breeds. Resting behavior exhibited an inverse trend to grazing time, with cattle resting more on low-quality pasture. Nguni cattle spent 31.2 % of their time resting on high-quality pasture, but this increased to 42.7 % when pasture quality declined (*p* = 0.009). Bonsmara cattle showed a similar pattern, increasing their resting time from 36.8 % to 48.3 %. The significant breed-by-environment interaction (*p* = 0.041) suggests that breed-specific adaptations to pasture quality influenced resting behavior. Walking time also increased when pasture quality declined. Nguni cattle walked more on low-quality pasture (9.3 %) compared to high-quality pasture (6.1 %) (*p* = 0.015), indicating increased movement in search of better forage. Bonsmara cattle showed a similar but slightly less pronounced increase from 6.9 % to 8.6 %. The significant interaction (*p* = 0.045) suggests that both breeds adjusted their movement patterns differently in response to pasture quality. Drinking time followed a similar trend, with cattle spending more time drinking on low-quality pasture, although this effect was more pronounced in Bonsmara cattle. Nguni cattle increased their drinking time from 2.0 % on high-quality pasture to 2.9 % on low-quality pasture (*p* = 0.028), while Bonsmara cattle showed a similar increase from 2.3 % to 3.4 %. However, the breed effect was not significant (*p* = 0.061), suggesting that both breeds responded similarly to changes in pasture quality in terms of water intake.

**Table 3:** Effect of pasture quality on grazing behavior.

Behavior Variable	Breed	High-Quality Pasture	Low-Quality Pasture	*p*-value (Breed)	*p*-value (Environment)	*p*-value (Breed × Environment)
Grazing Time (%)	Nguni	61.5 ± 3.2ᵃ	50.3 ± 2.7ᵇ	0.02	0.01	0.03
	Bonsmara	57.8 ± 2.9ᵃᵇ	47.5 ± 2.5ᵇ			
Resting Time (%)	Nguni	31.2 ± 1.8ᵃ	42.7 ± 2.1ᵇ	0.03	0.01	0.04
	Bonsmara	36.8 ± 1.9ᵃᵇ	48.3 ± 2.3ᵇ			
Walking Time (%)	Nguni	6.1 ± 0.6ᵃ	9.3 ± 0.8ᵇ	0.03	0.02	0.05
	Bonsmara	6.9 ± 0.7ᵃᵇ	8.6 ± 0.9ᵇ			
Drinking Time (%)	Nguni	2.0 ± 0.4ᵃ	2.9 ± 0.5ᵇ	0.06	0.03	0.07
	Bonsmara	2.3 ± 0.5ᵃᵇ	3.4 ± 0.6ᵇ			

Note: Values are expressed as Mean ± SE. Within each row, different superscripts (ᵃ,ᵇ) indicate significant differences (*p* < 0.05) between environmental conditions.

As shown in Table 4, Water availability had a significant impact on the grazing behavior of both Nguni and Bonsmara cattle. Under high water availability, Nguni cattle spent more time grazing (59.8 %) compared to when water was scarce (48.6 %) (*p* = 0.010). A similar trend was observed in Bonsmara cattle, where grazing time decreased from 54.1 % to 44.2 % under low water availability. This suggests that limited water access reduces grazing activity, likely due to increased time spent searching for water or conserving energy. Resting time followed an opposite pattern, with Nguni cattle resting significantly more under low water availability (9.9 %) compared to when water was abundant (6.8 %) (*p* = 0.019). Bonsmara cattle exhibited a similar response, though the change was less pronounced. Walking time also increased with reduced water availability, as Nguni cattle moved more (3.7 %) compared to when water was plentiful (2.4 %) (*p* = 0.022), while Bonsmara cattle showed a similar increase from 2.8 % to 3.9 %. The significant breed-by-environment interactions indicate that while both breeds adapted their behavior to water scarcity, their responses were not identical.

**Table 4:** Effect of water availability on grazing behavior.

Behavior Variable	Breed	High Water Availability	Low Water Availability	*p*-value (Breed)	*p*-value (Environment)	*p*-value (Breed × Environment)
Grazing Time (%)	Nguni	59.8 ± 3.0ᵃ	48.6 ± 2.6ᵇ	0.02	0.01	0.04
	Bonsmara	54.1 ± 2.7ᵃᵇ	44.2 ± 2.3ᵇ			
Resting Time (%)	Nguni	6.8 ± 0.7ᵃ	9.9 ± 1.0ᵇ	0.04	0.02	0.05
	Bonsmara	7.3 ± 0.8ᵃᵇ	9.0 ± 1.1ᵇ			
Walking Time (%)	Nguni	2.4 ± 0.5ᵃ	3.7 ± 0.6ᵇ	0.05	0.02	0.06
	Bonsmara	2.8 ± 0.6ᵃᵇ	3.9 ± 0.7ᵇ			

Note: Values are expressed as Mean ± SE. Within each row, different superscripts (ᵃ,ᵇ) indicate significant differences (*p* < 0.05) between environmental conditions.

As illustrated in Table 5, weather conditions significantly influenced the behavioral patterns of both Nguni and Bonsmara cattle. Under favorable weather conditions, Nguni cattle spent more time grazing (60.7 %) compared to when weather conditions were unfavorable (49.2 %) (*p* = 0.012). Similarly, Bonsmara cattle reduced their grazing time from 56.3 % in favorable weather to 45.8 % under unfavorable conditions. This decline in grazing activity suggests that harsh weather conditions may limit forage intake, possibly due to discomfort or increased energy expenditure for thermoregulation. Conversely, resting time increased under unfavorable weather, with Nguni cattle resting more (46.5 %) compared to when conditions were favorable (37.1 %) (*p* = 0.016). Bonsmara cattle showed a similar trend, with resting time rising from 40.3 % to 49.7 %. Walking time also increased as weather conditions worsened, with Nguni cattle walking more (8.8 %) than during favorable weather (6.3 %) (*p* = 0.021), while Bonsmara cattle exhibited a comparable increase from 7.0 % to 8.2 %. Drinking time followed the same pattern, increasing from 2.2 % to 3.5 % in Nguni cattle and from 2.7 % to 3.8 % in Bonsmara cattle, indicating a greater need for hydration under unfavorable weather (*p* = 0.029*). The significant breed-by-environment interactions highlight that while both breeds adjusted their behavior in response to weather conditions, their responses were not identical.

**Table 5:** Effect of weather conditions on behavioral variables of Nguni and Bonsmara Cattle.

Behavior Variable	Breed	Favorable Weather	Unfavorable Weather	*p*-value (Breed)	*p*-value (Environment)	*p*-value (Breed × Environment)
Grazing Time (%)	Nguni	60.7 ± 3.1ᵃ	49.2 ± 2.8ᵇ	0.03	0.01	0.04
	Bonsmara	56.3 ± 2.8ᵃᵇ	45.8 ± 2.4ᵇ			
Resting Time (%)	Nguni	37.1 ± 1.9ᵃ	46.5 ± 2.5ᵇ	0.03	0.02	0.05
	Bonsmara	40.3 ± 2.0ᵃᵇ	49.7 ± 2.6ᵇ			
Walking Time (%)	Nguni	6.3 ± 0.7ᵃ	8.8 ± 0.9ᵇ	0.04	0.02	0.05
	Bonsmara	7.0 ± 0.8ᵃᵇ	8.2 ± 0.9ᵇ			
Drinking Time (%)	Nguni	2.2 ± 0.5ᵃ	3.5 ± 0.6ᵇ	0.06	0.03	0.07
	Bonsmara	2.7 ± 0.6ᵃᵇ	3.8 ± 0.7ᵇ			

Note: Values are expressed as Mean ± SE. Within each row, different superscripts (ᵃ,ᵇ) indicate significant differences (*p* < 0.05) between environmental conditions.

### 3.4 Regression for behavioral variables

**Fig. 2:**
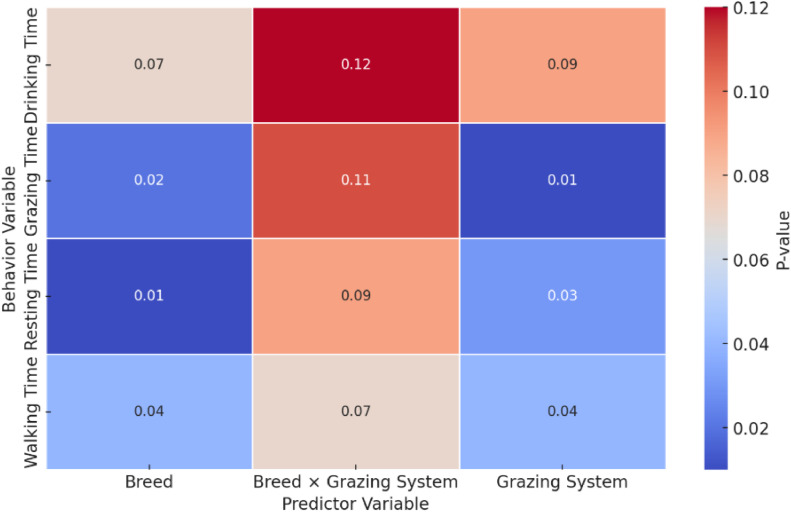
Heatmap illustrating regression *p*-values for behavioral variables.

Fig. 2 reveals significant predictors of grazing behavior among Nguni and Bonsmara cattle in different grazing systems. For grazing time, the breed of cattle significantly influenced the percentage of time spent grazing, with Nguni cattle grazing 4.8 % more than Bonsmara cattle (*B* = 4.8, SE = 1.9, *p* = 0.02). Additionally, the grazing system had a significant impact, with cattle in commercial systems grazing 5.3 % more than those in traditional systems (*B* = 5.3, SE = 2.0, *p* = 0.01). The interaction between breed and grazing system was not significant (*B* = 2.1, SE = 1.5, *p* = 0.11). Resting time was also significantly affected by breed and grazing system. Nguni cattle rested 6.2 % less than Bonsmara cattle (*B* = −6.2, SE = 2.1, *p* = 0.01), and cattle in commercial systems rested 4.8 % less than those in traditional systems (*B* = −4.8, SE = 2.2, *p* = 0.03). The interaction term between breed and grazing system was not significant (*B* = −1.6, SE = 1.7, *p* = 0.09). Walking time showed that Nguni cattle walked 0.9 % less than Bonsmara cattle (*B* = −0.9, SE = 0.4, *p* = 0.04). Cattle in commercial systems walked 0.8 % more than those in traditional systems (*B* = 0.8, SE = 0.5, *p* = 0.04). The interaction between breed and grazing system was not significant (*B* = −0.2, SE = 0.3, *p* = 0.07). For drinking time, there was a marginally significant trend where Nguni cattle drank 0.9 % more than Bonsmara cattle (*B* = 0.9, SE = 0.4, *p* = 0.07). The grazing system showed a non-significant trend with cattle in commercial systems drinking 0.4 % less than those in traditional systems (*B* = −0.4, SE = 0.5, *p* = 0.09). The interaction between breed and grazing system was not significant (*B* = −0.3, SE = 0.4, *p* = 0.12).

### 3.5 Correlations between forage quality, grazing time, and environmental factors

**Fig. 3:**
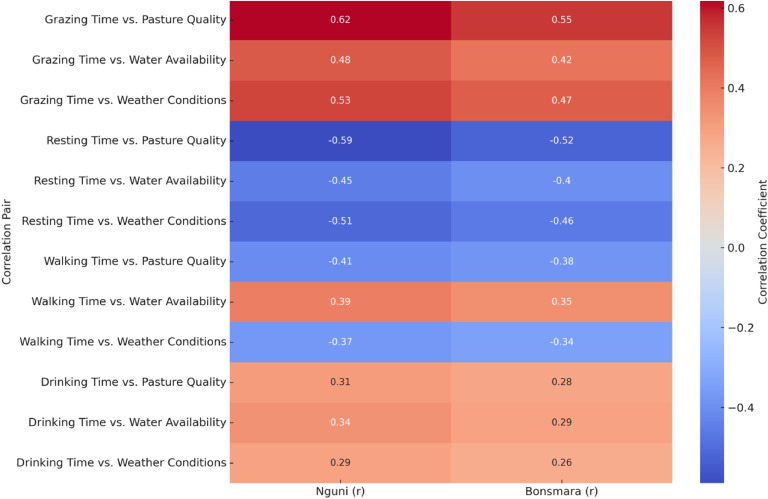
Heatmap illustrating correlations between forage quality, grazing time, and environmental factors.

Fig. 3 reveals significant relationships between grazing behaviors and environmental factors for both Nguni and Bonsmara cattle. Grazing time was positively correlated with pasture quality, water availability, and favorable weather conditions for both breeds. Specifically, Nguni cattle showed a stronger correlation with pasture quality (*r* = 0.62, *p* = 0.01) compared to Bonsmara cattle (*r* = 0.55, *p* = 0.02). Similarly, Nguni cattle's grazing time was significantly correlated with water availability (*r* = 0.48, *p* = 0.03) and weather conditions (*r* = 0.53, *p* = 0.02), while Bonsmara cattle exhibited slightly weaker but still significant correlations for these factors (*r* = 0.42, *p* = 0.04 for water availability and *r* = 0.47, *p* = 0.03 for weather conditions). Resting time showed negative correlations with pasture quality, water availability, and weather conditions. For Nguni cattle, resting time was significantly negatively correlated with pasture quality (*r* = −0.59, *p* = 0.01), water availability (*r* = −0.45, *p* = 0.03), and weather conditions (*r* = −0.51, *p* = 0.02). Bonsmara cattle displayed similar negative correlations: with pasture quality (*r* = −0.52, *p* = 0.02), water availability (*r* = −0.40, *p* = 0.04), and weather conditions (*r* = −0.46, *p* = 0.03). Walking time had weaker correlations with environmental factors compared to grazing and resting times. Nguni cattle's walking time showed a negative correlation with pasture quality (*r* = −0.41, *p* = 0.04) and weather conditions (*r* = −0.37, *p* = 0.05), while having a positive but weak correlation with water availability (*r* = 0.39, *p* = 0.05). Bonsmara cattle exhibited similar trends with slightly weaker correlations: negative with pasture quality (*r* = −0.38, *p* = 0.05) and weather conditions (*r* = −0.34, *p* = 0.06), and positive with water availability (*r* = 0.35, *p* = 0.06). Drinking time showed marginal correlations with the environmental factors. For Nguni cattle, drinking time had a weak positive correlation with pasture quality (*r* = 0.31, *p* = 0.07), water availability (*r* = 0.34, *p* = 0.06), and weather conditions (*r* = 0.29, *p* = 0.08). Bonsmara cattle showed similarly weak correlations: with pasture quality (*r* = 0.28, *p* = 0.08), water availability (*r* = 0.29, *p* = 0.07), and weather conditions (*r* = 0.26, *p* = 0.09).

## 4 Discussion

Cattle grazing dynamics are fundamental to ecosystem functioning and agricultural sustainability, particularly in diverse environments such as those found in Southern Africa (Améndola et al., 2020; Barbieri et al., 2014). Nguni and Bonsmara cattle, representing indigenous and commercial breeds, exhibit distinct genetic and adaptive traits shaped by natural selection and selective breeding (Slayi et al., 2023; Nyamushamba et al., 2017). Understanding their grazing behaviors across traditional communal rangelands and commercial ranches provides insights into how breed-specific traits and environmental conditions influence forage utilization and cattle productivity. The observed differences in grazing strategies between Nguni and Bonsmara cattle can be attributed to inherent genetic adaptations. Nguni cattle demonstrated extended foraging periods and a broader dietary range, consistent with their evolutionary adaptation to resource-limited environments. Their higher step rates and prolonged grazing duration suggest greater metabolic efficiency, which allows them to thrive in communal systems characterized by fluctuating forage availability (Katiyatiya et al., 2017; Owens et al., 2009, Singaravadivelan et al., 2023). Conversely, Bonsmara cattle, selectively bred for intensive beef production, exhibited a preference for high-quality forage, shorter grazing bouts, and higher weight gain in managed pastures. These findings align with previous studies emphasizing the superior growth performance of Bonsmara cattle under structured feeding conditions (Casey, 2021; Nyamushamba et al., 2017).

The semi-arid conditions of the study area posed significant constraints on forage availability, particularly during the dry season. Seasonal fluctuations in rainfall and temperature influenced grazing time, movement patterns, and bite rates, with the dry season imposing greater constraints on Bonsmara cattle in communal systems. Heat stress had a more pronounced effect on Bonsmara cattle, leading to reduced grazing activity and increased resting periods. In contrast, Nguni cattle exhibited greater resilience, maintaining consistent foraging activity under high temperatures, reinforcing their reputation for heat tolerance (Lancaster et al., 2021). These findings highlight the importance of incorporating heat mitigation strategies, such as rotational grazing with adequate shade and water provision, to optimize cattle productivity in varying climatic conditions. Forage selection varied significantly between breeds and grazing systems. Nguni cattle exhibited a broader dietary preference, consuming a mix of native grasses, shrubs, and herbs, particularly in communal systems. Their greater intake of legumes (25 % vs. 5 % in traditional systems) suggests an adaptive strategy to enhance protein intake through nitrogen-fixing plants such as *Medicago sativa* and *Stylosanthes* spp. (Parsons et al., 2020). The ability of Nguni cattle to efficiently utilize lower-quality forage may provide a competitive advantage in degraded rangelands with limited palatable grasses (Gusha et al., 2016). In contrast, Bonsmara cattle demonstrated a preference for improved grasses such as *Cenchrus ciliaris* and *Digitaria eriantha*, consistent with their selection for growth performance in structured grazing systems (Nyamushamba et al., 2017). Their reliance on high-quality forage indicates a potential need for supplementary feeding in nutrient-poor communal rangelands to sustain optimal productivity.

The results of this study underscore the importance of aligning grazing management strategies with breed-specific dietary needs. In communal systems with high forage variability, Nguni cattle may be better suited due to their flexible grazing strategies. Conversely, Bonsmara cattle may require targeted nutritional interventions to maintain productivity under extensive grazing conditions. These findings suggest that integrating adaptive management strategies, such as strategic supplementation and rotational grazing, could enhance livestock sustainability in diverse agroecological contexts. Further research is needed to explore the genetic basis of the observed differences between Nguni and Bonsmara cattle. Longitudinal studies assessing the long-term effects of grazing systems on cattle health and productivity would provide deeper insights into sustainable livestock management. Additionally, investigating microbiome differences between breeds across different grazing environments could enhance our understanding of forage conversion efficiency and digestive adaptability. Advanced technologies, including GPS tracking and remote sensing, could further refine behavioral and environmental data collection, providing more precise assessments of grazing dynamics. Future research integrating these approaches will be essential for developing more efficient and sustainable cattle production systems in Southern Africa.

### 4.2 Limitations and future research directions

Despite providing valuable insights into the behavioral responses of Nguni and Bonsmara cattle to environmental conditions, this study has several limitations. The research was conducted in a specific geographic region, which may limit the generalizability of findings to other climatic zones and grazing systems where variations in vegetation, altitude, and seasonal conditions could influence cattle behavior differently. Additionally, the study focused on a short observation period, potentially overlooking long-term behavioral adaptations that might emerge over different seasons. Another limitation is the lack of physiological measures, such as cortisol levels and metabolic markers, which could have provided a more comprehensive understanding of stress responses and adaptation mechanisms. While breed-level differences were analyzed, individual variations within breeds, influenced by factors such as age, previous exposure to stressors, and genetic predisposition, were not extensively considered. Furthermore, direct behavioral observations, though structured, may introduce observer bias, highlighting the need for automated tracking technologies such as GPS collars and accelerometers to enhance data accuracy. The study also did not account for potential influences of past management practices, such as controlled grazing or supplementation, which may affect behavioral responses. Additionally, social interactions within herds, such as dominance hierarchies and competition for resources, were not deeply analyzed, though these factors could significantly impact grazing behavior and resource utilization.

To build on these findings and address these limitations, future research should expand to diverse agro-ecological zones to enhance the applicability of the results across different environmental conditions. Longitudinal studies spanning multiple seasons or years would provide deeper insights into long-term behavioral adaptation strategies. Integrating physiological and behavioral data, including stress markers like heart rate and cortisol levels, could offer a more holistic understanding of cattle responses to environmental stressors. The use of advanced monitoring technologies, such as GPS tracking and motion sensors, would also improve data accuracy and reduce observer bias. Furthermore, investigating the genetic basis of breed-specific adaptations could inform selective breeding programs aimed at improving resilience to climatic stress. Future research should also examine the role of different livestock management strategies, including rotational grazing, supplementary feeding, and shade provision, to identify practical solutions for optimizing cattle performance. Additionally, assessing social interactions and herd dynamics could provide valuable insights into how behavioral hierarchies influence access to resources and overall adaptation in communal and commercial rangeland systems. By exploring these areas, future studies can contribute to more sustainable livestock management strategies, ensuring the resilience of cattle production systems under varying environmental conditions.

## 5 Conclusion

This study examines the complex relationship between breed characteristics and environmental factors in influencing the grazing behaviors of Nguni and Bonsmara cattle in both communal and commercial farming systems. The findings indicate that Nguni cattle demonstrate greater adaptability and foraging efficiency in communal rangelands, showcasing resilience to resource limitations and fluctuating environmental conditions. In contrast, Bonsmara cattle excel in intensive commercial systems, where they benefit from controlled management and superior pasture quality. These differences highlight the necessity of breed-specific management strategies to enhance productivity and sustainability. Additionally, key environmental factors such as pasture availability, water access, and climatic conditions, play a significant role in shaping cattle grazing patterns, emphasizing the need for targeted interventions to improve resource-use efficiency. Considering the increasing challenges posed by climate variability and land degradation, a more integrated approach to livestock management is vital. By improving pasture quality, optimizing water distribution, and aligning management practices with the specific needs of each breed, farmers can enhance productivity while preserving ecological balance. Future research should focus on long-term evaluations of breed-environment interactions under changing climatic conditions, utilizing advanced monitoring tools to refine adaptive management strategies. These initiatives will support the development of resilient and sustainable livestock production systems that prioritize both animal welfare and rangeland conservation.

## Funding

Financial support received from the National Research Foundation, grant number TS64 (UID: 99787), is acknowledged.

## Ethical clearance and approval

The study was conducted in compliance with the University of Fort Hare's Research Ethics Policy regarding the housing and treatment of animals. The research protocol underwent thorough review and received approval under the ethical clearance certificate number JAJ051SMPO01 from the institutional Animal Research Ethics Committee.

## Data availability statement

Data will be made available upon reasonable request.

## CRediT authorship contribution statement

**Mhlangabezi Slayi:** Writing – original draft, Visualization, Validation, Project administration, Methodology, Investigation, Formal analysis, Data curation, Conceptualization. **Ishmael Festus Jaja:** Writing – review & editing.

## Declaration of competing interest

The authors declare that they have no known competing financial interests or personal relationships that could have appeared to influence the work reported in this paper.

## References

[bib0001] Acocks J.P.H. (1988).

[bib0002] Améndola L., Solorio F.J., Ku-Vera J.C., Massioti R.D.A., Zarza H., Mancera K.F. (2020). A pilot study on the foraging behaviour of heifers in intensive silvopastoral and monoculture systems in the tropics. Animal : An International Journal of Animal Bioscience.

[bib0003] Asizua D. (2010).

[bib0004] Barbieri C.W., Quadros F.L.F., Jochims F., Soares É.M., Oliveira L.B., Carvalho R.M.R. (2014). Sward structural characteristics and performance of beef heifers reared under rotational grazing management on campos grassland. American Journal of Plant Sciences.

[bib0005] Cantalapiedra-Hijar G., Abo-Ismail M., Carstens G.E., Guan L.L., Hegarty R., Kenny D.A. (2018). Review: Biological determinants of between-animal variation in feed efficiency of growing beef cattle. Animal: An International Journal of Animal Bioscience.

[bib0006] Casey N.H. (2021). A profile of South African sustainable animal production and greenhouse gas emissions. Animal Frontiers.

[bib0007] Calus M.P.L., Perez B.C., de Vos J., Madsen O., Ayres L.L., Bovenhuis H. (2023). Abstracts ISAG 2023 39th international society for animal genetics conference.

[bib0008] Cavallini D., Raspa F., Marliani G., Nannoni E., Martelli G., Sardi L. (2023). Growth performance and feed intake assessment of Italian holstein calves fed a hay-based total mixed ration: Preliminary steps towards a prediction model. Veterinary Sciences.

[bib0009] Eastern Cape Socio-Economic Consultative Council (ECSECC) (2012). http://www.ecsecc.org/files/library/documents/EasternCape_withDMs.pdf.

[bib0010] Fayemi P.O., Muchenje V. (2012). Meat in African context: From history to science. African Journal of Biotechnology.

[bib0011] Fitzsimons C., Kenny D.A., Fahey A.G., McGee M. (2014). Feeding behavior, ruminal fermentation, and performance of pregnant beef cows differing in phenotypic residual feed intake offered grass silage. Journal of Animal Science.

[bib0012] Fitzsimons C., Kenny D.A., Deighton M.H., Fahey A.G., McGee M. (2013). Methane emissions, body composition, and rumen fermentation traits of beef heifers differing in residual feed intake. Journal of Animal Science.

[bib0013] Gusha J., Chiuta T., Katsande S., Zvinorova P.I., Kagande S.M. (2016). Performance of cattle reared on rangelands supplemented with farm-formulated diets during the dry season in Zimbabwe. Animal Production Science.

[bib0014] Hardy M.B., Walker L.S. (1991). Determining sample size for assessing species composition in grassland. Journal of the Grassland Society of Southern Africa.

[bib0015] Jochims F., Soares ÉM.O.de, Kuinchtner L.B., Casanova B.C., Marin P.T. (2020). Timing and duration of observation periods of foraging behavior in natural grasslands. Frontiers in Veterinary Science.

[bib0016] Jorge-Smeding E., Bonnet M., Renand G., Taussat S., Graulet B., Ortigues-Marty I. (2021). Common and diet-specific metabolic pathways underlying residual feed intake in fattening Charolais yearling bulls. Scientific Reports.

[bib0017] Katiyatiya C.L.F., Bradley G., Muchenje V. (2017). Thermotolerance, health profile and cellular expression of HSP90AB1 in Nguni and Boran cows raised on natural pastures under tropical conditions. Journal of Thermal Biology.

[bib0018] Kelly A.K., McGee M., Jr Crews DH, Fahey A.G., Wylie A.R., Kenny D.A (2010). Effect of divergence in residual feed intake on feeding behavior, blood metabolic variables, and body composition traits in growing beef heifers. Journal of Animal Science.

[bib0019] Lamy E., van Harten S., Sales-Baptista E., Guerra M.M.M., de Almeida A.M. (2012). Environmental stress and amelioration in livestock production.

[bib0020] Lancaster P.A., Davis M.E., Rutledge J.J., Cundiff L.V. (2021). Relationships among feed efficiency traits across production segments and production cycles in cattle. Translational animal science.

[bib0021] Lawrence P., Kenny D.A., Earley B., McGee M. (2012). Grazed grass herbage intake and performance of beef heifers with predetermined phenotypic residual feed intake classification. Animal : An International Journal of Animal Bioscience.

[bib0022] Linde D.A. (2018).

[bib0023] Mapfumo L., Muchenje V., Mupangwa J.F., Scholtz M.M. (2017). Changes in biochemical proxy indicators for nutritional stress resilience from Boran and Nguni cows reared in dry arid rangeland. Tropical Animal Health and Production.

[bib0024] Mapiye O., Chikwanha O.C., Makombe G., Dzama K., Mapiye C. (2020). Livelihood, food and nutrition security in Southern Africa: What role do indigenous cattle genetic resources play?. Diversity.

[bib0025] Mapiye C., Chikwanha O.C., Chimonyo M., Dzama Kennedy (2019). Strategies for sustainable use of indigenous cattle genetic resources in Southern Africa. Diversity.

[bib0026] Matlebyane M.M., Ng'ambi J.W.W., Aregheore E.M. (2009). Relationships between chemical composition and in vitro digestibility of some common forage species used for ruminant livestock production in three chief areas of Capricorn Region, Limpopo Province, South Africa. Research Journal of Agriculture and Biological Sciences.

[bib0027] McDonnell R.P., Hart K.J., Boland T.M., Kelly A.K., McGee M., Kenny D.A. (2016). Effect of divergence in phenotypic residual feed intake on methane emissions, ruminal fermentation, and apparent whole-tract digestibility of beef heifers across three contrasting diets. Journal of Animal Science.

[bib0028] Meyer A.M., Kerley M.S., Kallenbach R.L. (2008). The effect of residual feed intake classification on forage intake by grazing beef cows. Journal of Animal Science.

[bib0029] Mezzalira J.C., Bremm C., Trindade J.K., Nabinger C., Carvalho P.C.F. (2012). The ingestive behaviour of cattle in large-scale and its application to pasture management in heterogeneous pastoral environments. Journal of Agricultural Science and Technology.

[bib0030] Moya D., Holtshausen L., Marti S., Gibb D.G., McAllister T.A., Beauchemin K.A. (2014). Feeding behavior and ruminal pH of corn silage, barley grain, and corn dried distillers' grain offered in a total mixed ration or in a free-choice diet to beef cattle. Journal of Animal Science.

[bib0031] Muchenje V., DzamaChimonyo K.M., Chimonyo M., Raats J.G., Strydom P.E. (2008). Meat quality of Nguni, Bonsmara and Aberdeen angus steers raised on natural pasture in the Eastern Cape, South Africa. Meat Science.

[bib0032] Mucina L., Rutherford M.C. (2011).

[bib0033] Musemwa L., Muchenje V., Mushunje A., Zhou L. (2012). The impact of climate change on livestock production amongst the resource-poor farmers of third world countries: A review. Asian Journal of Agriculture and Rural Development.

[bib0034] Myburgh J., Osthoff G., Hugo A., De Wit M., Nel K., Fourie D. (2012). Comparison of the milk composition of free-ranging indigenous African cattle breeds. South African Journal of Animal Science.

[bib0035] Napolitano F., Girolami A., Pacelli C., Braghieri A. (2011). Activity budgets and forage selection of podolian cattle, a semi wild Bovine breed. International Scholarly Research Network.

[bib0036] Nciizha A.D., Wakindiki I.I.C. (2012). Particulate organic matter, soil texture and mineralogy relations in some Eastern cape ecotopes in South Africa. South African Journal of Plant and Soil.

[bib0037] Nyamushamba G.B., Mapiye C., Tada O., Halimani T.E., Muchenje V. (2017). Conservation of indigenous cattle genetic resources in Southern Africa's smallholder areas: Turning threats into opportunities - A review. Asian-Australasian Journal of Animal Sciences.

[bib0038] Owens D., McGee M., Boland T., O'Kiely P. (2009). Rumen fermentation, microbial protein synthesis, and nutrient flow to the omasum in cattle offered corn silage, grass silage, or whole-crop wheat. Journal of Animal Science.

[bib0039] Parsons I.L., Johnson J.R., Kayser W.C., Tedeschi L.O., Carstens G.E. (2020). Characterization of feeding behavior traits in steers with divergent residual feed intake consuming a high-concentrate diet. Journal of Animal Science.

[bib0040] Scholtz M.M., Maiwashe A., Neser F.W.C., Theunissen A., Olivier W.J., Mokolobate M.C. (2013). Livestock breeding for sustainability to mitigate global warming, with the emphasis on developing countries. South African Journal of Animal Science.

[bib0041] Singaravadivelan A., Sachin P.B., Harikumar S., Vijayakumar P., Vindhya M.V., Farhana F.B. (2023). Life cycle assessment of greenhouse gas emission from the dairy production system. Tropical Animal Health and Production.

[bib0042] Slayi M., Kayima D., Jaja I.F., Mapiye C., Dzama K. (2023). Enteric methane output and weight accumulation of Nguni and Bonsmara cows raised under different grazing conditions. Pastoralism : Research, Policy and Practice.

[bib0043] Slayi M., Zhou L., Njisane Y.Z. (2022). Grass composition and distribution patterns as determinants of behavioral activities and weight accumulation of Nguni and Boran cattle post-relocation. Frontiers in Veterinary Science.

[bib0044] Slayi M., Njisane Y.Z., Muchenje V. (2021). Behavioural and haemato-biochemical responses of Nguni and Boran steers post relocation and herd regrouping in a novel environment. Journal of Applied Animal Welfare Science.

[bib0045] Trindade J.K., Pinto C.E., Neves F.P., Mezzalira J.C., Bremm C., Genro T.C.M. (2012). Forage allowance as a target of grazing management: Implications on grazing time and forage searching. Rangeland Ecology & Management.

[bib0046] Williams L.R., Jackson E.L., Hurley G.J., Swain D.L (2016). Drinking frequency effects on the performance of cattle: A systematic review. Journal of Animal Physiology and Animal Nutrition.

